# Dermatopontin, A Novel Adipokine Promoting Adipose Tissue Extracellular Matrix Remodelling and Inflammation in Obesity **†**

**DOI:** 10.3390/jcm9041069

**Published:** 2020-04-09

**Authors:** Xabier Unamuno, Javier Gómez-Ambrosi, Beatriz Ramírez, Amaia Rodríguez, Sara Becerril, Víctor Valentí, Rafael Moncada, Camilo Silva, Javier Salvador, Gema Frühbeck, Victoria Catalán

**Affiliations:** 1 Metabolic Research Laboratory, Clínica Universidad de Navarra, 31008 Pamplona, Spain; xunamuno@unav.es (X.U.); jagomez@unav.es (J.G.-A.); bearamirez@unav.es (B.R.); arodmur@unav.es (A.R.); sbecman@unav.es (S.B.); 2 CIBER Fisiopatología de la Obesidad y Nutrición (CIBEROBN), Instituto de Salud Carlos III, 31008 Pamplona, Spain; vvalenti@unav.es (V.V.); rmoncada@unav.es (R.M.); csilvafr@unav.es (C.S.); jsalvador@unav.es (J.S.); 3 Obesity and Adipobiology Group. Instituto de Investigación Sanitaria de Navarra (IdiSNA) 31008 Pamplona, Spain; 4 Department of Surgery, Clínica Universidad de Navarra, 31008 Pamplona, Spain; 5 Department of Anesthesia, Clínica Universidad de Navarra, 31008 Pamplona, Spain; 6 Department of Endocrinology & Nutrition, Clínica Universidad de Navarra, 31008 Pamplona, Spain

**Keywords:** dermatopontin, inflammation, obesity, fibrosis, adipose tissue, extracellular matrix remodeling

## Abstract

Compelling evidence suggests that dermatopontin (DPT) regulates collagen and fibronectin fibril formation, the induction of cell adhesion and the prompting of wound healing. We aimed to evaluate the role of DPT on obesity and its associated metabolic alterations as well as its impact in visceral adipose tissue (VAT) inflammation and extracellular matrix (ECM) remodelling. Samples obtained from 54 subjects were used in a case-control study. Circulating and VAT expression levels of DPT as well as key ECM remodelling- and inflammation-related genes were analysed. The effect of pro- and anti-inflammatory mediators on the transcript levels of DPT in visceral adipocytes was explored. The impact of DPT on ECM remodelling and inflammation pathways was also evaluated in cultured adipocytes. We show that obesity and obesity-associated type 2 diabetes (T2D) increased (*p* < 0.05) circulating levels of DPT. In this line, DPT mRNA in VAT was increased (*p* < 0.05) in obese patients with and without T2D. Gene expression levels of DPT were enhanced (*p* < 0.05) in human visceral adipocytes after the treatment with lipopolysaccharide, tumour growth factor (TGF)-β and palmitic acid, whereas a downregulation (*p* < 0.05) was detected after the stimulation with interleukin (IL)-4 and IL-13, critical cytokines mediating anti-inflammatory pathways. Additionally, we revealed that DPT increased (*p* < 0.05) the expression of ECM- (*COL6A3, ELN, MMP9, TNMD*) and inflammation-related factors (*IL6, IL8, TNF*) in human visceral adipocytes. These findings provide, for the first time, evidence of a novel role of DPT in obesity and its associated comorbidities by influencing AT remodelling and inflammation.

## 1. Introduction

During obesity, stiff matrix components are deposited in the adipose tissue (AT) extracellular matrix (ECM) modifying tissue biomechanics and tensile properties from its microenvironment. These abnormalities in the ECM structure disrupt the AT homeostasis promoting fibrosis, and hence, a reduction in the degree of plasticity of the ECM preventing AT from expanding in a healthy manner and leading to adverse metabolic consequences [1,2]. Moreover, obesity-associated low-grade chronic inflammation has been also proposed as a crucial promoter of changes in the ECM remodelling and composition of the AT [1]. Therefore, the study of molecules that can modulate the ECM is of significant interest, especially during obesity. Our group previously showed differentially-expressed genes involved in the immune function and inflammation pathways as well as in angiogenesis in the omental AT from patients with obesity obtained from microarray data [3]. Among these factors, we found an upregulation of dermatopontin (DPT), a 22-kDa protein with multiple physiological properties including collagen and fibronectin fibril formation, the induction of cell adhesion and the prompting of wound healing [4,5,6].

DPT, also known as tyrosine rich acidic matrix protein (TRAMP), is a noncollagenous protein of the ECM firstly discovered in the dermis [7]. DPT is clearly detected in several organs but the skin is considered to be the richest source. The relative richness of DPT (approximately 12–15 mg per kilogram of wet dermis weight) in the ECM suggests essential biological roles for this molecule [8,9]. DPT increases collagen fibrillogenesis but also reduces the collagen fibril thickness, the same as many other ECM molecules including decorin, lumican and fibromodulin [8]. Studies performed in *Dpt*-knockout mice also highlight the relevance of DPT in the regulation of ECM structure and fibrosis [10,11]. *Dpt*-null mice exhibited an Ehlers-Danlos syndrome phenotype characterised by fragile and elastic skin, a reduction in the accumulation of collagen in the ECM and an abnormal arrangement of collagen microfibrils [5,11,12]. Although no significant differences in anthropometric and metabolic characteristics were found in *Dpt*^−/−^ mice compared with their wild type counterparts, *Dpt* deficiency protected the liver from chemically- and oxidative stress-induced fibrosis, inhibiting collagen deposition [10,11]. In this line, *DPT* expression has been shown to be upregulated in the liver of patients with nonalcoholic steatohepatitis (NASH) and fibrosis with its expression decreasing after gastric bypass in parallel with the reduction of the fibrosis stage [10].

The ECM not only provides a mechanical support for the great variety of cells that constitute the AT but also regulates physiological processes through a variety of signalling pathways [13,14]. In this sense, known functions of DPT include the binding to the cell surface receptor integrin α3β1 and syndecan-1 as well as the interaction and modulation of the activity of transforming growth factor (TGF)-β, decorin and fibronectin [15]. Considering the functional characteristics of TGF-β, DPT has been described to enhance its biological activity [16]. Reportedly, DPT is a potent cell adhesion molecule in diverse cell types including keratinocytes, fibroblasts and endothelial cells [17,18]. DPT has also been involved in inhibiting cell proliferation such as in murine C2C12 myoblasts, keratinocytes, osteosarcoma MG-63 cells or papillary thyroid cancer cells [4,19]. Moreover, DPT has been found in wound fluids as well as in the provisional matrix, a term to describe factors that appear coincident with epidermal cell migration during skin wound healing [4]. Taken together, these results suggest that DPT mediates communication between the ECM environments being directly involved in the wound healing process.

Although compelling evidence suggests that DPT regulates collagen fibrillogenesis, the role of DPT in regulating ECM remodelling and inflammation in VAT in a context of obesity remains unknown. Owing to the multiple roles of DPT during wound healing and ECM reassembly, we hypothesised a regulatory role of DPT in ECM remodelling and inflammation in human visceral adipocytes. Therefore, we first analysed whether obesity and its associated pathologies T2D and nonalcoholic fatty liver disease (NAFLD) influence the expression levels of *DPT*. In addition, the regulation of *DPT* by different inflammatory mediators was further explored in human visceral adipocytes.

## 2. Materials and Methods

### 2.1. Patient Selection

Blood and tissue samples were obtained from 54 volunteers attending the Departments of Endocrinology & Nutrition and Surgery at the Clínica Universidad de Navarra. Clinical assessment including medical history and physical examination as well as the evaluation of comorbidities and body composition analysis were performed by a multidisciplinary team. Body mass index (BMI) was calculated as weight in kilograms divided by the square of height in meters and total body fat (BF) was estimated by air-displacement-plethysmography (Bod-Pod^®^, Life Measurements, Concord, CA, USA). Normal-weight was defined as a BMI ≤ 25 kg/m^2^ and obesity as a BMI ≥ 30 kg/m^2^. The waist-to-hip ratio (WHR) was determined as the quotient between the circumference of the waist (at the midway level between the lowest palpable rib and the iliac crest) and the hip (around the widest portion of the trochanters). Patients with obesity were further subclassified into two groups (normoglycaemia (NG) or T2D) according to the criteria of the Expert Committee on the Diagnosis and Classification of Diabetes [20]. Patients with severe systemic disease not related to obesity, infectious/inflammatory diseases, cancer, liver disease or severe nephropathy, pharmacological treatments, pregnancy or lactation as well as patients with serious eating disorders and people whose freedom is under legal or administrative requirement were excluded. The study was approved, from an ethical and scientific standpoint, by the Clínica Universidad de Navarra’s Ethical Committee responsible for research (approval number 2017.126) and the written informed consent of participants was obtained.

Visceral adipose tissue (VAT) samples were collected from patients undergoing either Nissen fundoplication (for hiatus hernia repair in normal-weight volunteers) or Roux-en-Y gastric bypass (RYGB) (for the treatment of morbid obesity in obese volunteers) at the Clínica Universidad de Navarra. Additionally, a liver biopsy was obtained from the patients with obesity during the bariatric surgery to establish a histological diagnosis of the hepatic state as well as for the study of gene expression. Since this procedure is not clinically justified in lean subjects, liver samples were only obtained from patients with obesity. The diagnosis of NAFLD was established following the criteria of Kleiner and Brunt by an expert pathologist masked to all the results of the assays [21]. VAT and liver samples were immediately frozen in liquid nitrogen and stored at −80 °C for subsequent analyses.

### 2.2. Analytical Measurements

Blood samples were drawn after an overnight fasting. Glucose was analysed by an automated analyser (Hitachi Modular P800, Roche, Basel, Switzerland) and insulin was determined by an enzyme-amplified chemiluminescence assay (IMMULITE^®^, Diagnostic Products Corp., Los Angeles, CA, USA) with intra- and interassay coefficients of variation of 4.2% and 5.7%, respectively. The HOMA and QUICKI indices were calculated to examine insulin resistance and sensitivity, respectively. Lipid profile (triglycerides, total cholesterol, high-density lipoprotein cholesterol (HDL) and low-density lipoprotein cholesterol (LDL)) was evaluated as previously described [22,23]. Hepatic enzymes were measured by enzymatic tests in an automated analyser (Roche/Hitachi Modular P800) and the AST/ALT ratio was calculated as an indirect indicator of fatty liver disease [24]. The concentrations of the inflammatory markers fibrinogen, high-sensitivity C-reactive protein (CRP), homocysteine and von Willebrand factor antigen (vWF) were analysed as previously reported [22,23]. A double-antibody RIA method (Linco Research, Inc., St. Charles, MO) was used to determine leptin concentrations. The intra- and interassay coefficients of variation were 5.0% and 4.5%, respectively [25]. DPT levels were determined by a commercially available ELISA kit (Cusabio, Wuhan, China) with intra- and interassay coefficients of variation being 8% and 10%, respectively.

### 2.3. RNA Isolation and Real-Time PCR

Tissue samples were homogenised with an Ultra-Turrax^®^ T25 basic (IKA^®^ Werke Gmbh, Staugen, Germany) and RNA isolation was performed using QIAzol^®^ Reagent (Qiagen, Hilden, Germany) for VAT and adipocytes and TRIzol^®^ Reagent (Invitrogen, Carlsbad, CA, USA) for liver biopsies. The RNeasy Mini Lipid Kit (Qiagen) in VAT and adipocytes and the RNeasy Mini kit (Qiagen) in liver biopsies were used for RNA purification. All samples were treated with DNase I (RNase Free DNase set, Qiagen). RNA (2 μg) were reverse transcribed to cDNA according to standard procedures [26]. The mRNA levels for adiponectin (*ADIPOQ*), collagen (*COL*)-*1A1*, *COL6A3*, decorin (*DCN*), elastin (*ELN*), *IL1B*, *IL6*, *IL8*, *IL18*, kruppel-like factor 4 (*KLF4*), matrix metalloproteinase (*MMP*)-2, *MMP9*, *TGFB1*, tumour necrosis factor-α (*TNF*) and tenomodulin (*TNMD*) were quantified by Real-Time PCR (7300 Real Time PCR System, Applied Biosystems, Foster City, CA, USA) as previously described [26]. Primers and probes (Merck, Darmstadt, Germany) were designed using the software Primer Express 2.0 (Applied Biosystems, Foster City, CA, USA) (Supplementary Table S1). The cDNA was amplified as previously described [26]. The endogenous control gene *18S* rRNA (Applied Biosystems) was the endogenous control for Real-Time PCR experiments and a relative quantification was obtained using the ΔΔCt formula. Relative gene expression was expressed as fold expression over the calibrator sample (average of gene expression corresponding to the normal-weight group or unstimulated adipocytes) [26]. Triplicates and positive and negative controls were included in all reactions.

### 2.4. Human Adipocyte Culture and Treatment

Human stromovascular fraction cells (SVFC) were isolated from VAT from patients with obesity as previously reported [27]. SVFC were seeded at 2 × 10^5^ cells/well and grown in adipocyte medium supplemented with 10% newborn calf serum (NCS). After 4 days, the medium was changed to adipocyte medium supplemented with 3% NCS, 0.5 mmol/L 3-isobutyl-1-methylxanthine (IBMX), 0.1 µmol/L dexamethasone, 1 µmol/L BRL49653 and 10 µg/mL insulin. Differentiated adipocytes (10th day of differentiation) were serum-starved for 24 h and then incubated with increasing concentrations of DPT (1, 10 and 100 ng/mL) (R & D systems, Minneapolis, MN), IL-4 (10, 100 and 1000 ng/mL) (R & D systems), IL-13 (10, 100 and 1000 ng/mL) (R & D systems), LPS (10, 100 and 1000 ng/mL) (Sigma), palmitic acid (100 and 200 nmol/L) (Sigma), TNF-α (1, 10 and 100 ng/mL) (Sigma) and TGF-β (1, 10 and 20 ng/mL) (R & D systems) for 24 h.

### 2.5. Statistical Analysis

Data are presented as mean ± standard error of the mean (SEM). Due to their nonparametric distribution, gene expression levels and C-reactive protein concentrations were logarithmically transformed. The other variables followed a normal distribution that was suitable for the application of parametric tests. Differences between groups were assessed by one-way ANOVA followed by Tukey’s or Dunnet’s post hoc tests as appropriate. Statistical analysis comparing gene expression levels of *DPT* and *TGFB* in liver as well as in VAT from patients with obesity classified according to suffering or not NAFLD was performed by two-tailed unpaired Student’s *t* tests. The relation between variables was evaluated by simple correlation (Pearson’s correlation coefficients (*r*)). Statistical analysis was performed using the SPSS version 15.0 statistical package (SPSS, Chicago, IL, USA). Levels of statistical significance were set at *p* value < 0.05.

## 3. Results

### 3.1. Circulating Levels of DPT are Increased in Obesity

Characteristics of the volunteers enrolled in the study are shown in Table 1. As expected, markers of adiposity were significantly higher (*p* < 0.001) in both groups of patients with obesity compared with lean volunteers. Patients with T2D were more insulin-resistant than lean and obese NG subjects, as evidenced by the increased concentrations of glucose (*p* < 0.001) and lower QUICKI index (*p* < 0.001). Noteworthy, individuals with obesity showed higher (*p* < 0.01) circulating levels of the inflammatory markers CRP and fibrinogen. Homocystein concentrations were significantly increased (*p* < 0.01) in patients with obesity and T2D. No differences were observed in the circulating levels of AST and ALP, but patients with T2D exhibited increased (*p* < 0.01) concentrations of ALT and γ-GT together with a decrease (*p* < 0.001) of the ratio AST/ALT.

**Figure 1:**
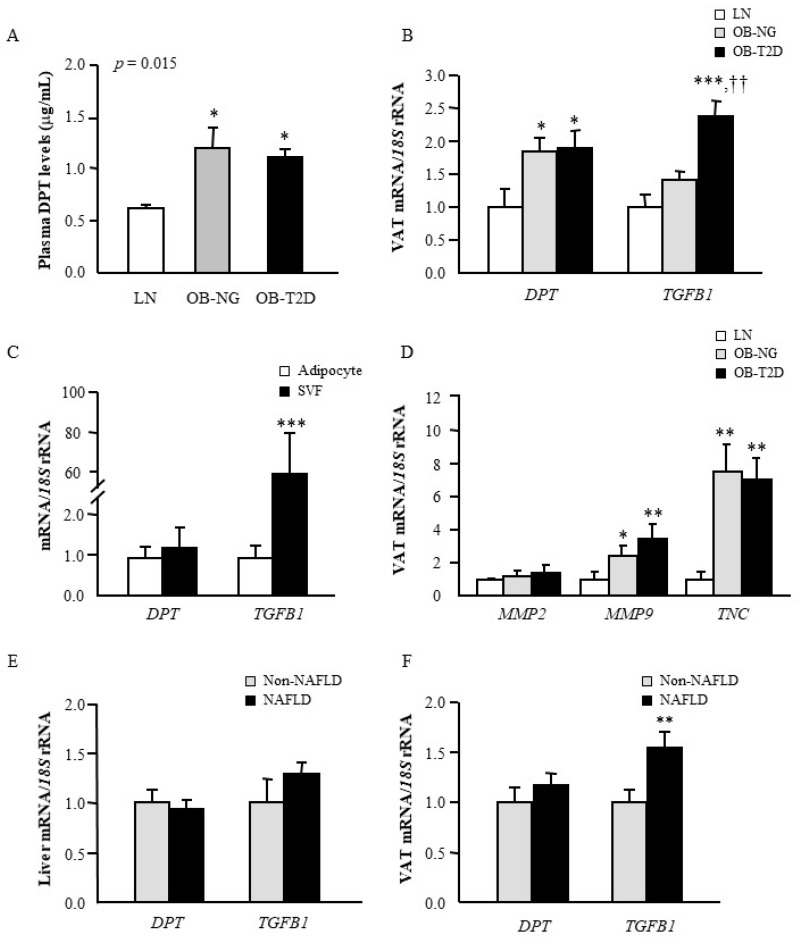
Circulating concentrations and gene expression levels of dermatopontin (DPT) in obesity, obesity-associated type 2 diabetes (T2D) and non-alcoholic fatty liver disease (NAFLD). (**A**) Fasting plasma concentrations of DPT in lean (LN) volunteers, obese normoglycemic (NG) subjects and obese patients with T2D. (**B**) Bar graphs show the mRNA levels of *DPT* and tumour growth factor-β (*TGFB1*) in visceral adipose tissue (VAT) from lean volunteers, obese NG subjects and obese patients with T2D as well as in (**C**) adipocytes and stromovascular fraction (SVF) cells. (**D**) Bar graphs show the mRNA levels of metalloproteinase (*MMP*)-2, *MMP9* and tenascin C (*TNC*) in VAT from lean volunteers, obese NG subjects and obese patients with T2D Gene expression levels of *DPT* and *TGFB1* in liver (**E**) and VAT (**F**) from obese volunteers classified according to the presence or not of NAFLD. Bars represent the mean ± SEM. Differences between groups were analysed by one-way ANOVA followed by Tukey’s tests as well as by paired or unpaired two-tailed Student’s *t* tests, where appropriate. * *p* < 0.05, ** *p* < 0.01 and *** *p* < 0.001 vs. LN subjects, adipocytes or nonNAFLD. ^††^ *p* < 0.01 vs. obese NG volunteers.

Significant differences in circulating DPT concentrations among the experimental groups were found (*p* = 0.015) to be significantly increased in both groups of patients with obesity as compared to normal-weight volunteers (Figure 1A). Differences remained statistically significant after adjustment by age and sex (*p* = 0.010 and *p* = 0.002, respectively). In this regard, a significant positive correlation (*p* < 0.001) was observed between circulating DPT concentrations and all anthropometric measurements as well as with insulin levels (*p* = 0.005) and HOMA index (*p* = 0.032), whereas a negative correlation with HDL-cholesterol (*p* = 0.001) was found (Table 2). Importantly, a negative association between plasma DPT levels and the marker of liver fibrosis AST/ALT was also observed (*p* = 0.019) (Table 2).

### 3.2. Obesity and Obesity-Associated T2D Increase Gene Expression Levels of DPT in VAT

Since plasma levels of DPT are increased in patients with obesity and considering the contribution of VAT to obesity-associated inflammation and metabolic alterations, we analysed mRNA levels of *DPT* together with those of *TGFB1* in this fat depot. Results showed higher gene expression levels of *DPT* (*p* < 0.01) in VAT in obesity and obesity-associated T2D (Figure 1B). In this sense, *DPT* mRNA levels were significantly associated with WHR (*r* = 0.31; *p* = 0.028). Moreover, gene expression levels of *TGFB* were significantly upregulated (*p* < 0.01) in obese patients with T2D compared to normal-weight and obese NG volunteers (Figure 1B). Notably, gene expression levels of *DPT* were positively correlated with mRNA levels of *TGFB1* (*r* = 0.41; *p =* 0.002). Macrophages, fibroblasts and myofibroblasts are known as important sources of DPT. However, the expression of DPT by other cell types is poorly understood. To validate which cell type preferentially contributed to the expression levels of *DPT* in VAT, adipocytes and SVFC were isolated from VAT samples obtained from patients with obesity. Whereas expression levels of *TGFB1* were highly upregulated (*p* < 0.0001) in SVFC compared to mature adipocytes, no differences in the mRNA levels of *DPT* were found (Figure 1C). We also detected an increase in the gene expression levels of the ECM-related genes *MMP9* and *TCN* in both groups of patients with obesity compared with normal-weight subjects (Figure 1D). Although mRNA levels of *MMP2* were higher in patients with obesity, differences were not statistically significant (*p* = 0.078). However, a positive association between the expression of *DPT* and *MMP2* was found (*r* = 0.30; *p* = 0.026).

Given that the liver represents a main metabolic organ and that one of the best-recognized hepatic disorders related with obesity is NAFLD, our aim was to investigate the hepatic regulation of *DPT* and *TGFB1* in this condition. No differences were detected between obese patients with or without NAFLD in the expression levels of *DPT* and *TGFB1* in the liver (Figure 1E), while *TGFB1* gene expression was significantly upregulated (*p* < 0.01) in obese patients with NAFLD in VAT (Figure 1F). Remarkably, a strong correlation (*r* = 0.56; *p* < 0.001) was found between mRNA levels of *DPT* and *TGFB1* in the liver. We also observed a negative association (*r* = –0.33; *p* = 0.009) between gene expression of *TGFB1* in the liver and the ratio AST/ALT.

### 3.3. Effect of Inflammatory and Anti-Inflammatory Factors in DPT Expression in Visceral Adipocytes

**Figure 2:**
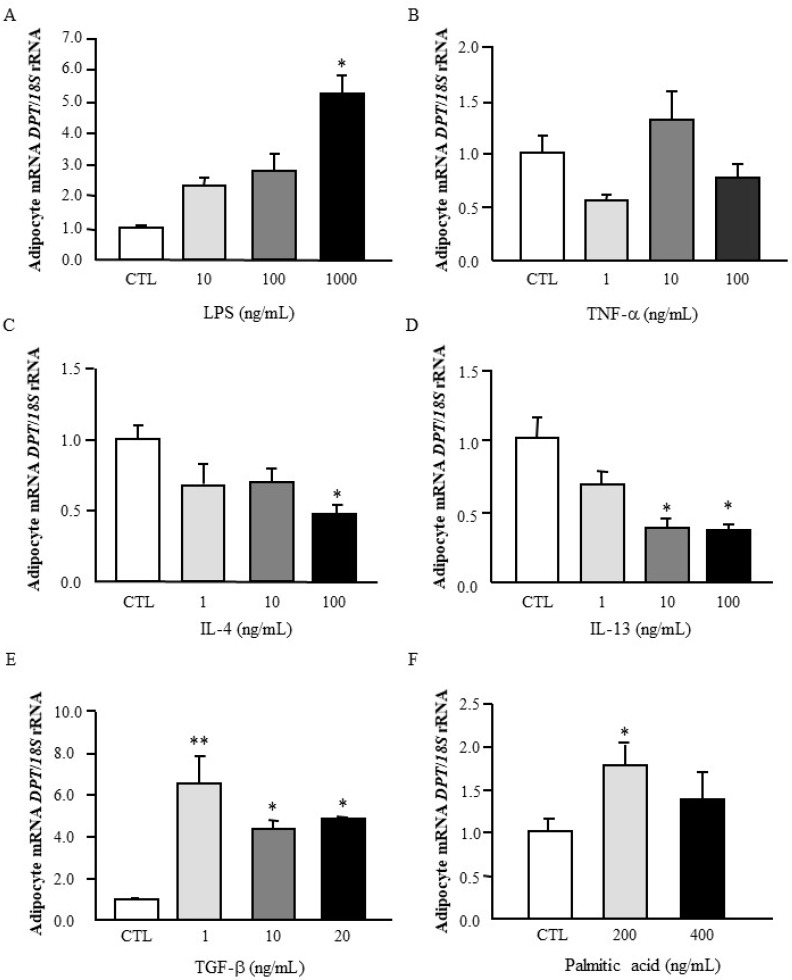
Effect of inflammation-related factors on mRNA levels of *DPT*. Gene expression levels of *DPT* in cultured human visceral adipocytes incubated during 24 h with (**A**) LPS, (**B**) TNF-α, (**C**) IL-4, (**D**) IL-13, (**E**) TGF-β and (**F**) palmitic acid. Gene expression levels in unstimulated cells were assumed to be 1. Values are the mean ± SEM (*n* = 6 per group). Differences between groups were analysed by one-way ANOVA followed by Dunnett’s tests. * *p* < 0.05 and ** *p* < 0.01 vs. unstimulated cells.

Since unresolved inflammation and the inappropriate ECM remodelling are related processes that reflect a dysfunctional AT, human omental adipocytes were stimulated with different inflammation-related mediators to analyse their influence on *DPT* expression. As shown in Figure 2A, *DPT* mRNA levels were induced at the highest dose (*p* < 0.05) by LPS, a well-known exogenous inflammatory factor that initiates acute inflammatory responses, but no differences were found after the treatment with TNF-α, an endogenous inflammatory factor (Figure 2B). Oppositely, a significant downregulation (*p* < 0.05) of mRNA levels of *DPT* after the treatment with the anti-inflammatory cytokines IL-4 and IL-13 was observed in human visceral adipocytes (Figure 2C,D). TGF-β has been shown to enhance the expression of DPT on normal skin cultured fibroblasts [28]. Importantly, we also found an upregulation (*p* < 0.05) of *DPT* expression after the treatment with TGF-β in visceral adipocytes (Figure 2E). In vivo and in vitro studies have revealed that a chronic exposure to palmitate, a saturated free fatty acid, is associated with the induction of fibrosis [29,30]. In this sense, we found that palmitic acid enhanced (*p* < 0.05) the mRNA levels of *DPT* in adipocytes (Figure 2F).

### 3.4. Dermatopontin Upregulates ECM Remodelling and Inflammation-Related Genes in Visceral Adipocytes

**Figure 3:**
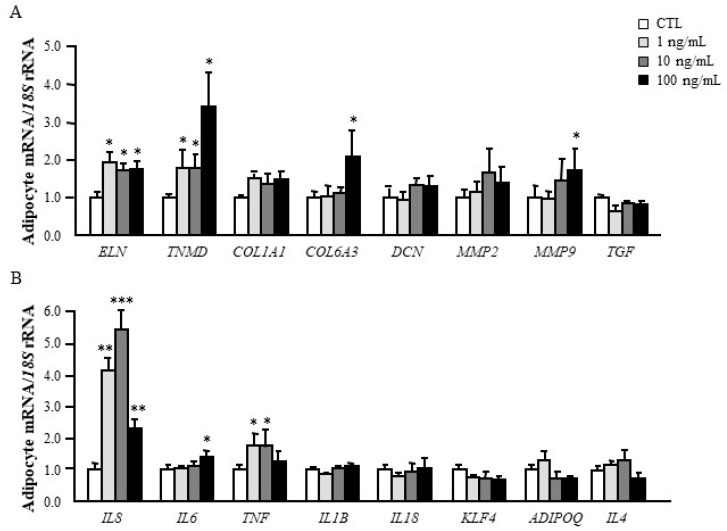
DPT induces the expression of ECM- and inflammation-related factors in human visceral adipocytes. Gene expression levels of extracellular matrix remodelling-related molecules (**A**) as well as inflammatory factors (**B**) in human visceral adipocytes stimulated with recombinant DPT (1, 10 and 100 ng/mL) for 24 h. Gene expression levels in unstimulated cells were assumed to be 1. Values are the mean ± SEM (*n* = 6 per group). Differences between groups were analysed by one-way ANOVA followed by Dunnet’s post hoc tests. * *p* < 0.05, ** *p* < 0.01 and *** *p* < 0.001 vs. unstimulated cells.

It is known that DPT acts as a potential contributor to liver fibrosis increasing collagen deposition [8]. Therefore, we assessed whether DPT can modify the expression of genes involved in the ECM remodelling and inflammatory response in human visceral adipocytes. Cells were stimulated with increasing concentrations of DPT for 24 h. As shown in Figure 3A, DPT treatment significantly upregulated (*p* < 0.05) the expression levels of genes closely related to ECM structure and remodelling (*COL6A3*, *ELN*, *MMP9* and *TNMD*). Unexpectedly, no effect of the DPT treatment was found in the modulation of *TGFB1* and *DCN*. We also detected an increase (*p* < 0.05) in the expression of the well-recognized inflammatory markers *IL6*, *IL8* and *TNF* (Figure 3B).

## 4. Discussion

Alterations in the ECM composition of the AT can aberrantly activate signalling pathways that promote the development of obesity-associated comorbidities [31]. The excess deposition of main components of the ECM during obesity including structural proteins (collagens) and different types of adhesion proteins (fibronectin, laminin, elastins and proteoglycans) triggers not only a physical restriction on AT expansion but also the necrosis of adipocytes, inflammation and metabolic dysfunction [31,32]. In this sense, the study was designed to ascertain the role of DPT, a small protein with significant properties in collagen fibril formation, in regulating ECM remodelling and inflammation in VAT in the context of obesity. To our knowledge, this is the first study reporting that obesity and obesity-associated T2D increase circulating levels of *DPT* as well as its gene expression levels in VAT. In addition, we revealed that inflammation-related factors regulate mRNA levels of *DPT*. Consistently, we further unveiled the role of DPT in the regulation of ECM remodelling and inflammation in human visceral adipocytes.

The obesity-associated ECM remodelling of AT is greatly linked with elevated circulating levels of ECM proteins or ECM-derived peptides in parallel with increased levels of inflammatory cytokines. In the present study we report, for the first time, that circulating concentrations of DPT are increased in obese patients with and without T2D. A previous study also identified the presence of DPT in serum, although its relative amount was much lower than in the wound fluid [4]. The detection of DPT in the serum was unexpected and its function is not well-established. It has been suggested that DPT could function as a coagulation factor, since its invertebrate homologue acts as an agglutination factor [33]. We also found a positive association between circulating concentrations of DPT and all anthropometric measurements strengthening its involvement in obesity. Importantly, we demonstrated a positive correlation between DPT and insulin levels and HOMA, suggesting a link between DPT and insulin sensitivity. Increased levels of cholesterol in Dpt*** mice have been described [11] and accordingly, a negative association of DPT with HDL-cholesterol levels was detected in our study.

Tissue-specific distribution of *Dpt* has been previously evaluated in different mouse tissues, exhibiting a major expression in skin, adipose tissues and lungs [10]. The increased mRNA expression levels of *DPT* in VAT from obese patients compared to normal-weight volunteers is a novel finding, and this is the first observation that DPT may play a role in the VAT dysfunction in obesity. Although *Dpt*-knockout mice did not show clear anatomical abnormality or gross metabolic alterations, they showed an increased skin elasticity, a significant decrease in the relative thickness of the dermis and an impaired collagen fibrillogenesis [10,11]. These data indicate the critical role of DPT in collagen accumulation [11]. Recently, *DPT* was found associated with the degree of fibrosis in human liver disease with its hepatic expression being significantly decreased after gastric bypass [10]. The increased levels of *DPT* in the VAT from patients with obesity suggest a role of this protein in fibrogenesis, resulting in increased ECM deposition and hence, altering the ECM composition and favouring fibrosis. Reportedly, DPT interacts and increases TGF-β signalling [16] and, in this sense, we observed increased levels of *TGFB1* mRNA in VAT from obese patients with T2D as well as a positive association of gene expression levels of *DPT* and *TGFB1*. TGF-β is a proangiogenic cytokine considered a master regulator of fibrosis in AT [34,35]. TGF-β increases the expression of multiple ECM components such as collagens, fibronectin and matrix metalloproteinases [13]. Our results indicate that TGF-β upregulated the expression levels of *DPT* in human visceral adipocytes. *DPT* expression was described as being inducible by TGF-β in the human stellate LX2 cell line as well as in normal skin cultured fibroblasts [10,28]. In accordance with previous results in fibroblasts [28], the anti-inflammatory factors IL-4 and IL-13 reduced the mRNA of *DPT* also in visceral adipocytes. Moreover, LPS and palmitic acid, both potential candidates in the development of AT inflammation, increased the expression of *DPT*. Adipocytes have been proposed as a crucial site for the response to LPS, one of the most potent exogenous inflammatory factors. Indeed, mature adipocytes are recognized as a key source of LPS-induced cytokines that are essential for the setting of the obesity-associated low-grade inflammation. We observed a stimulatory effect of LPS on the expression of *DPT*, suggesting the possible role of DPT in the inflammatory pathways associated to obesity. Therefore, our results suggest that the obesity-associated inflammation in AT enhances the expression of *DPT*.

The multiple effects of DPT in cell adhesion and proliferation as well as in ECM remodelling have been studied in different cell types but its role in adipocytes remains unknown. We found a significant increase in the expression of relevant ECM components (*COL6A3*, *ELN*, *MMP9*, *TNMD*) in adipocytes after DPT treatment. Collagens constitute the most abundant structural components of the ECM and compared to other components, collagen VI is the most highly expressed in mature adipocytes exhibiting an important metabolic role [13]. Moreover, expression of *COL6* is increased in the VAT of patients with obesity [36,37]. We found that DPT increased the expression of *COL6A3* in adipocytes, thereby stimulating transduction pathways involved in profibrotic responses as well as in the capacity of adipocytes to expand. We also found increased levels of *ELN*, another ECM protein found in almost all tissues, after DPT stimulation. Elastin-derived peptides have been shown to promote insulin resistance by inhibiting glucose uptake in AT and muscle [38]. Similar to COL6, elastin deposition is increased in VAT depots with obesity [39]. Moreover, *ELN* expression is downregulated in *Col6*-knockout mice which exhibited a less dense collagen matrix that allows adipocyte hypertrophy [13]. *MMP* expression in AT is significantly increased in patients with obesity, suggesting that these enzymes are involved in ECM remodelling during obesity [40,41]. Consistently, an increased expression of *MMP9* in VAT was detected in patients with obesity and the treatment with DPT also increased its expression in adipocytes. The functions of MMPs during fibrosis are not limited to the degradation and turnover of most components of the ECM, they also influence cellular proliferation and survival, control different aspects of inflammation, activate fibroblasts and play a role in the control of adipocyte differentiation [40,42,43]. Specifically, MMP-9 has been associated with inflammation and fibrosis, mainly through the synthesis and deposition of collagen I in reactive fibrosis and its ability to activate TGF-β [44]. We also analysed the effects of DPT in the expression of *TNMD* due to its high and main expression in human AT [45] and because *Tnmd*-knockout mice showed disorganised collagen fibrils [46]. We detected an upregulation of TNMD after the treatment with DPT. A role of TNMD in promoting healthy AT expansion has been speculated since mice overexpressing *Tnmd* in AT displayed improved insulin sensitivity [47]. In this sense, the higher *TNMD* expression detected in insulin-resistant patients in the omental fat has been proposed as a compensatory mechanism to increase adipocyte replenishment in this condition [47]. However, the exact role of TNMD in ECM remodelling in obesity remains unknown. Unexpectedly, no differences in *DCN* and *TGFB1* were found after DPT treatment, although it has been reported that DPT stimulates the expression of *TGFB1* in other cell types and a tight association with decorin has been proposed [8,48].

AT fibrosis is associated with a stiff ECM inhibiting adipocyte expansion, promoting the necrosis of adipocytes and, finally, an enhanced inflammatory response. We evaluated the role of DPT in the expression of different inflammation-related factors showing an upregulation of *IL6*, *IL8* and *TNF*. In this sense, loss of *Dpt* expression in mice triggers an increase in the expression of genes involved in the *Clec7a/Dectin-1* pathway, responsible for suppressing Toll-like receptor (TLR)-4 signalling pathways and therefore favouring anti-inflammatory responses [10,49].

DPT is a noncollagenous protein with important biological functions in the ECM. We described for the first time that obesity and obesity-associated T2D increase circulating levels of DPT. We also report for the first time that *DPT* gene expression levels are increased in the VAT from patients with obesity as well as the role of DPT in the regulation of ECM remodelling and inflammation in human visceral adipocytes. Taken together, we proposed that DPT exerts an important role in the pathogenesis of ECM remodelling and inflammation during obesity and its associated comorbidities. Modulation of DPT as well as other ECM remodelling proteases and signalling molecules that mediate adipocyte-ECM interactions constitute promising pharmacological targets to improve obesity-associated comorbidities. However, further studies are needed to understand the exact role of DPT in orchestrating ECM remodelling in AT.

## Figures and Tables

**Table 1:** Anthropometric and biochemical characteristics of subjects included in the study.

	Lean	Obese NG	Obese T2D
***n* (males, females)**	13 (5, 8)	21 (6, 15)	20 (8, 12)
**Age (years)**	38 ± 2	41 ± 3	42 ± 3
**BMI (kg/m^2^)**	21.8 ± 0.9	41.6 ± 1.3 **	46.5 ± 1.3 ***^,††^
**Body fat (%)**	22.6 ± 2.1	53.2 ± 1.4 ***	52.1 ± 2.1 ***
**Waist circumference (cm)**	75 ± 3	119 ± 3 ***	129 ± 2 ***^,†^
**Hip circumference (cm)**	94 ± 1	127 ± 2 ***	136 ± 3 ***^,†^
**Waist-to-hip ratio**	0.79 ± 0.02	0.94 ± 0.02 ***	0.96 ± 0.02 ***
**Neck circumference (cm)**	34 ± 1	38 ± 1 **	42 ± 1 **^,††^
**Fasting glucose (mg/dL)**	85 ± 6	86 ± 2	123 ± 6 **^,†††^
**2h OGTT glucose (mg/dL)**	–	112 ± 3	196 ± 14 ^†††^
**Fasting insulin (** **µU/mL)**	7.2 ± 1.3	17.1 ± 1.4	22.2 ± 3.8
**2h OGTT insulin (** **µU/mL)**	–	72.7 ± 8.6	140.0 ± 18.1 ^†††^
**HOMA**	1.6 ± 0.4	4.0 ± 1.1	6.3 ± 1.3
**QUICKI**	0.369 ± 0.016	0.334 ± 0.010	0.305 ± 0.007 **
**Triglycerides (mg/dL)**	67 ± 12	97 ± 7	143 ± 13 **^,††^
**Cholesterol (mg/dL)**	171 ± 8	195 ± 10	189 ± 7
**LDL-cholesterol (mg/dL)**	88 ± 5	119 ± 7	113 ± 6
**HDL-cholesterol (mg/dL)**	69 ±	56 ± 5	47 ± 3 *
**Leptin (ng/mL)**	8.9 ± 2.4	58.2 ± 5.9 ***	40.8 ± 8.4 **
**CRP (mg/L)**	0.53 ± 0.08	9.0 ± 2.1 ***	8.1 ± 1.8 ***
**Fibrinogen (mg/dL)**	194 ± 12	402 ± 18 ***	354 ± 23 ***
**von Willebrand factor (%)**	57 ± 10	143 ± 15 **	131 ± 14 **
**Homocysteine (** **µmol/L)**	6.6 ± 0.5	9.4 ± 0.8	10.4 ± 0.6 **
**AST (U/L)**	12 ± 1	18 ± 4	16 ± 4
**ALT (U/L)**	6 ± 3	22 ± 3	28 ± 2 **
**AST/ALT**	2.24 ± 0.14	0.87 ± 0.08 ***	0.67 ± 0.06 ***
**ALP (U/L)**	74 ± 6	90 ± 6	92 ± 7
**γ-GT (U/L)**	13 ± 3	19 ± 3	30 ± 5

ALP, alkaline phosphatase; ALT, alanine aminotransferase; AST, aspartate aminotransferase; BMI, body mass index; CRP, C-reactive protein; **γ**-GT, **γ**-glutamyltransferase; HOMA, homeostatic model assessment; NG, normoglycemic; OGTT, oral glucose tolerance test; QUICKI, quantitative insulin sensitivity check index; T2D, type 2 diabetes. Data are mean ± SEM. CRP concentrations were logarithmically transformed for statistical analysis. Differences between groups were analysed by one-way ANOVA followed by Tukey’s post hoc tests or by unpaired two-tailed Student’s *t* tests, where appropriate. **p < 0.05, ***p < 0.01 and ****p < 0.001 vs. lean. ^†^ *p* < 0.05, ^††^ *p* < 0.01 and ^†††^ *p* < 0.01 vs. obese NG.

**Table 2:** Univariate analysis of the correlations between circulating levels of dermatopontin (DPT) with anthropometric measurement and biochemical parameters.

	Circulating Levels of DPT	
	*r*	*p*
**Age**	0.17	0.224
**BMI**	0.44	**<0.001**
**BF**	0.35	**0.009**
**Waist circumference**	0.53	**<0.001**
**Hip circumference**	0.36	**0.008**
**Waist-to-hip ratio**	0.53	**<0.001**
**Neck circumference**	0.49	**<0.001**
**Fasting glucose**	0.01	0.934
**Fasting insulin**	0.43	**0.005**
**HOMA**	0.34	**0.032**
**QUICKI**	–0.26	0.106
**Triglycerides**	0.31	0.052
**Cholesterol**	0.18	0.262
**LDL-cholesterol**	0.05	0.741
**HDL-cholesterol**	–0.49	**<0.001**
**Leptin**	0.27	0.122
**CRP**	0.06	0.706
**Fibrinogen**	0.01	0.933
**von Willebrand factor**	0.16	0.357
**Homocystein**	0.46	**0.006**
**AST**	0.09	0.555
**ALT**	0.02	0.907
**AST/ALT**	–0.36	0.019
**ALP**	0.09	0.549
**γ-GT**	0.02	0.882

ALP, alkaline phosphatase; ALT, alanine aminotransferase; AST, aspartate aminotransferase; BMI, body mass index; CRP, C-reactive protein; γ-GT, γ-glutamyltransferase; DPT, dermatopontin; HOMA, homeostatic model assessment; QUICKI, quantitative insulin sensitivity check index. Differences between groups were analysed by Pearson’s correlation coefficients. Bold figures highlight statistically significant differences.

## Supplementary Materials

The following are available online at https://www.mdpi.com/2077-0383/9/4/1069/s1, Table S1: Sequences of the primers and TaqMan^®^ probes.

## Abbreviations

ADIPOQ, adiponectin; ALP, alkaline phosphatase; ALT, alanine aminotransferase; ARG1, arginase 1; AST, aspartate aminotransferase; AT, adipose tissue; BMI, body mass index; COL, collagen, CRP, C-reactive protein; DBP, diastolic blood pressure; DCN, decorin; DPT, dermatopontin; ECM, extracellular matrix; ELN, elastin; γ-GT, γ-glutamyltransferase; HOMA, homeostasis model assessment; IL, interleukin; KLF4, kruppel-like factor 4; LPS, lipopolysaccharide; MMP, matrix metalloproteinase; NAFLD nonalcoholic fatty liver disease; NG, normoglycaemic; OGTT, oral glucose tolerance test; QUICKI, quantitative insulin sensitivity check index; RYGB, Roux-en-Y gastric bypass; SBP, systolic blood pressure; SVFC, stroma-vascular fraction cells; TGFB1, tumour growth factor β; TNF, tumour necrosis factor-α; TNMD, tenomodulin; T2D, type 2 diabetes; VAT, visceral adipose tissue.
