# Supplementary material for: Dermatopontin, A Novel Adipokine Promoting Adipose Tissue Extracellular Matrix Remodelling and Inflammation in Obesity
Source: J Clin Med. 2020 Apr 9;9(4):1069. doi: 10.3390/jcm9041069 (PMC7230369; doi:10.3390/jcm9041069)
Supplement: Supplementary file 1 [file jcm-09-01069-s001.pdf]

Supplemental Table 1. Sequences of the primers and TaqMan® probes.

Gene (GenBank accession)	Oligonucleotide sequence (5'-3')
ADIPOQ (NM_001177800)	
Forward	GGAGATCCAGGTCTTATTGGTCCTA
Reverse	CCTTGGATTCCCGGAAAGC
TaqMan® Probe	FAM-ACATCGGTGAAACCGGAGTACCCGG-TAMRA
COL1A1 (NM_000088.3)	
Forward	CTCCCGGGCCTCAAGGTAT
Reverse	TTGCTCCAGAGGGACCTTGTT
TaqMan® Probe	FAM-TCTTCCTGGCCCCTCTGGTGAACCT-TAMRA
COL6A3 (NM_004369.3)	
Forward	GACGGAGATCTGGCTGATTTACA
Reverse	AGATGCATTAGCCGCTCCAA
TaqMan® Probe	FAM-AGAACCTCCGCCAAGAAGGAGTCCGT-TAMRA
DCN (NM_001920.5)	
Forward	AGAAGCTCTCCTACATCCGCATT
Reverse	CTGCATCAACTCTGCTGATTTGT
TaqMan® Probe	FAM-TTCCTCAAGGTCTTCCTCCTTCCCTTACG-TAMRA
DPT (NM_001937.5)	
Forward	GGCAGTTTTACTGTTGTCGCTACA
Reverse	CATGTCCATTTCCTCACCATAGTG
TaqMan® Probe	FAM-TGCCCATATTCCTGCTGGCTAACAACAG-TAMRA
ELN (NM_000501.4)	
Forward	TGGAGGAGTGGCAGCAAGA
Reverse	CTTCCGGCCACAAGCTTTC
TaqMan® Probe	FAM-TCGGATTGTCTCCCATTTTCCCAGGT-TAMRA
IL1B (NM_000576)	
Forward	CAGTGGCAATGAGGATGACTTG
Reverse	GTAGTGGTGGTCGGAGATTCGTA
TaqMan® Probe	FAM-TGGCCCTAAACAGATGAAGTGCTCCTTCC-TAMRA
IL6 (NM_000600)	
Forward	GCCCTGAGAAAGGAGACATGTAAC
Reverse	ATCCATCTTTTTCAGCCATCTTTG
TaqMan® Probe	FAM-AGGCACTGGCAGAAAACAACCTGAACC-TAMRA
IL8 (NM_000584.3)	
Forward	ACCTTTCCACCCCAAATTTATCA
Reverse	TTCTCAGCCCTCTTCAAAAACTTC
TaqMan® Probe	FAM-CCACACTGCGCCAACACAGAAATTATTGTA-TAMRA
KLF4 (NM_001314052.1)	
Forward	ACCTACACAAAGAGTTCCCATCTCA
Reverse	GTTTACGGTAGTGCCTGGTCAGTT

TaqMan® Probe	FAM-CCTGCGAACCCACACAGGTGAGAAA-TAMRA
MMP2 (NM_004530)	
Forward	CCATTTTGATGACGATGAGCTATG
Reverse	GTTGTACTCCTTGCCATTGAACAA
TaqMan® Probe	FAM-CTTGGGAGAAGGCCAAGTGGTCCGT-TAMRA
MMP9 (NM_004994)	
Forward	GCCCGGACCAAGGATACAGT
Reverse	CCCCTCAGTGAAGCGGTACA
TaqMan® Probe	FAM-ACGCGCTGGGCTTAGATCATTCCTCA-TAMRA
TGFB (NM_000660)	
Forward	GCCCAGCATCTGCAAAGC
Reverse	TCCTTGCGGAAGTCAATGTACA
TaqMan® Probe	FAM-CACCAACTATTGCTTCAGCTCCACGGA-TAMRA
TNC (NM_002160)	
Forward	AGGCGATCCCAGACAGTCAGT
Reverse	TCCAGCTGACAGTAGCCGAATT
TaqMan® Probe	FAM-TCCAGCTGACAGTAGCCGAATT-TAMRA
TNF (NM_000594)	
Forward	CCCCAGGGACCTCTCTCTAATC
Reverse	ACATGGGCTACAGGCTTGTCA
TaqMan® Probe	FAM-CCTCTGGCCCAGGCAGTCAGATCAT-TAMRA
TNMD (NM_022144.2)	
Forward	GGCCCTAACTCTAATTGTCCTGTTT
Reverse	TTCTGGTCACAGGATCAATTTCC
TaqMan® Probe	FAM-CCGGAGGTACCCAAAAAAGCCTATGACA-TAMRA

ADIPOQ, adiponectin; *COL*, collagen; *DCN*, decorin; *DPT*, dermatopontin; *ELN*, elastin; *IL*, interleukin; *KLF4*, kruppel-like factor 4; *MMP*, matrix metalloproteinase; *TGFB1*, transforming growth factor-β; *TNC*, tenascin C; *TNF*, tumor necrosis factor-α; *TNMD*, tenomodulin.
